# The Government and Physical Deterioration

**Published:** 1905-07-29

**Authors:** 


### The Government and Physical Deterioration.

The Presidential Address delivered by Lord Londonderry to the Royal Institute of Public Health may be assumed to be an authoritative statement of the view taken by the Government of the questions which have been brought to the front by the Report of the Committee on Physical Deterioration, and by the discussions, in and out of Parliament, which that report has occasioned. If this be so, it indicates a policy which, in the language of a now obsolete form of legal pleading, might be described as one of confession and avoidance. Lord Londonderry made no attempt to deny, to explain away, or to minimise, the conditions which the report described; but he contended that they were not to be corrected by legislation, and advised as his remedy "the creation of a large-hearted sentiment of public interest in place of timorous counsels and sectional prejudice." These, no doubt, are brave words; but it would be extremely difficult to attach to them any definite meaning, unless it be that the Government will certainly do nothing, and that philanthropists are at full liberty to do whatever they please. As Sydney Smith once put the matter, A sees B in distress, and thinks it is the duty of C to relieve him. The same papers which reported the address reported also a speech by Lord Roberts on the establishment of rifle clubs in schools, in which he said that the scheme was suspended between the War Office and the Education Department, each maintaining that it was in the province of the other, and neither doing anything for its promotion. Lord Londonderry announced that the Board of Education had given very careful consideration to the teaching of hygiene to young children, and intended shortly to "issue certain suggestions as to the kind of teaching which was advisable;" but he had previously stated that the whole question of the power of sanitary authorities, or of any public authorities, in regard to individual citizens was beset with great difficulties, and some of the proposals which had been made for the purpose of increasing the powers of these authorities appeared to import new matters of principle into our legislation. He was not at present prepared to give any support to such proposals. In other words, Lord Londonderry, presumably as the spokesman of the Government, totally ignores the radical change which has been effected in the position of the child by the enactment which requires that he shall be educated at the public expense. By virtue of this enactment he ceases to be the private property of his parents, and the State clearly becomes entitled to see that he is dealt with in such a manner that his education shall not be wholly unprofitable either to himself or to the community. This implies that he shall be sufficiently fed and clothed, and that he shall be brought up in reasonably satisfactory hygienic surroundings; and it involves the principle that parents shall not only be restrained from gross cruelty or from manifestly injurious neglect, but that they shall be compelled to meet the State half way, and shall be held responsible for the proper discharge of their duties to the children whom they have seen fit to bring into the world. Such compulsion as this cannot be exercised by philanthropy, however effectively it may be organised. Drunkenness, idleness, rags, filth, when displayed by parents, should be promptly and sharply punished, and those guilty of them should be made to understand that parentage entails duties to the community and to the children which the law will not suffer to be evaded. In so far as such defaults were the necessary consequences of unavoidable poverty, they might safely be left to the various benevolent organisations which would certainly spring into existence in order to cope with them; but it is well known, to all who are conversant with the lives and the moral standards of the lowest sections of the community, that poverty is more often an excuse than a reason. When the children are dirty, or starving, or ragged, there is still money for the publican, for the football match, or for the racecourse.

In the meanwhile, if the Education Board be really desirous to initiate an effective reform in the education of the industrial classes, it may find a basis of sound principles in those two paragraphs of the Church Catechism which deal with the question of *duty*. It is the custom to-day to talk proudly of the great Admiral who made duty his watchword; but we sometimes forget the secret of its efficacy. The men who fought at Trafalgar had been taught to regard the performance of duty as one of the chief ends of life, as a condition the fulfilment of which enabled the young to face danger and death with composure, and the old to repeat the *Nunc dimittis* with content. In place of the "duty towards God" learnt by our grandfathers, the children of to-day are taught to repeat subtleties of metaphysical theology; and, in the place of their duty towards their neighbours, they learn the precepts of the trades union. It requires but a slender acquaintance with facts in order to perceive that the deterioration of large sections of our population is not physical alone, and that more than physical remedies are required in order to cope with it effectively. It may be that the right remedies will be discovered, and that time will be afforded for their effective application. What is certain is that the moral and the physical health of the community are closely interwoven, and that the conditions favourable to the one will be favourable also to the other. The physical health of the inhabitants of our great cities has been suffered to deteriorate, very largely because so-called "authorities" have not exercised the powers which have been entrusted to them by the Legislature for the protection of their neighbours. Why should the Municipality of Lincoln be permitted to cultivate typhoid fever, or the Municipality of Leicester to cultivate small-pox? It is an elementary duty of Government to protect the lives and property of the governed; and this duty is not fulfilled by transferring it to bodies which are certain to leave it undone. To compel them to employ their powers might be a new principle in English legislation, but it would be one fraught with unspeakable advantage to future generations.

